# Supplementary figures and images for: Defining the Rhythm: Developing a New Method to Describe Tremor and Myoclonus
Source: Mov Disord. 2025 Sep 9;40(12):2654–63. doi: 10.1002/mds.70034 (PMC12710117; doi:10.1002/mds.70034)

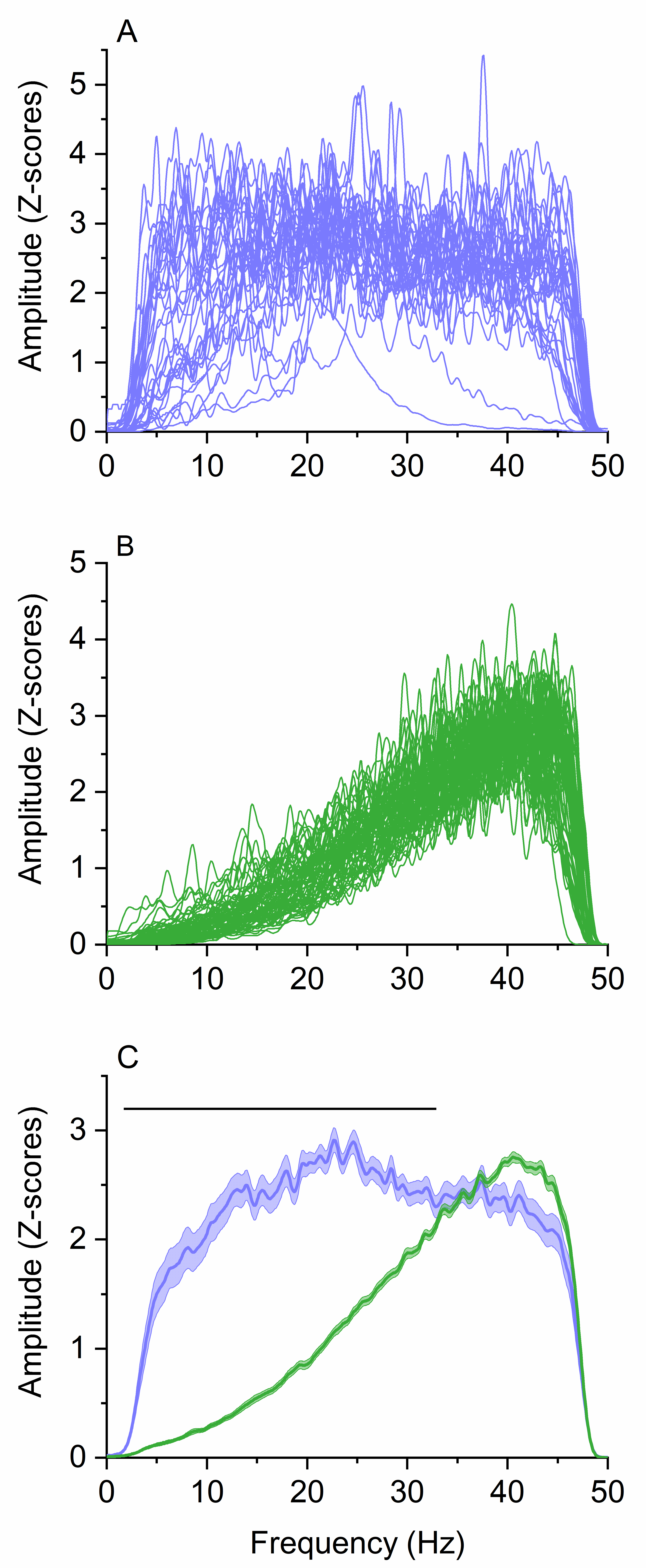

Supplement: Supplementary file 1 — Figure S1. (A, B) Power spectral densities (PSDs) of individual subjects in the myoclonus and healthy control groups, respectively. For visual comparison, voltage values were converted to Z‐scores and baseline offsets were removed. Each line represents a single subject. (C) Group‐averaged PSDs (blue: myoclonus; green: healthy controls), with shaded areas indicating the standard error of the mean. The horizontal black line marks the frequency range in which a statistically significant difference between groups was identified. [file MDS-40-2654-s005.tif]

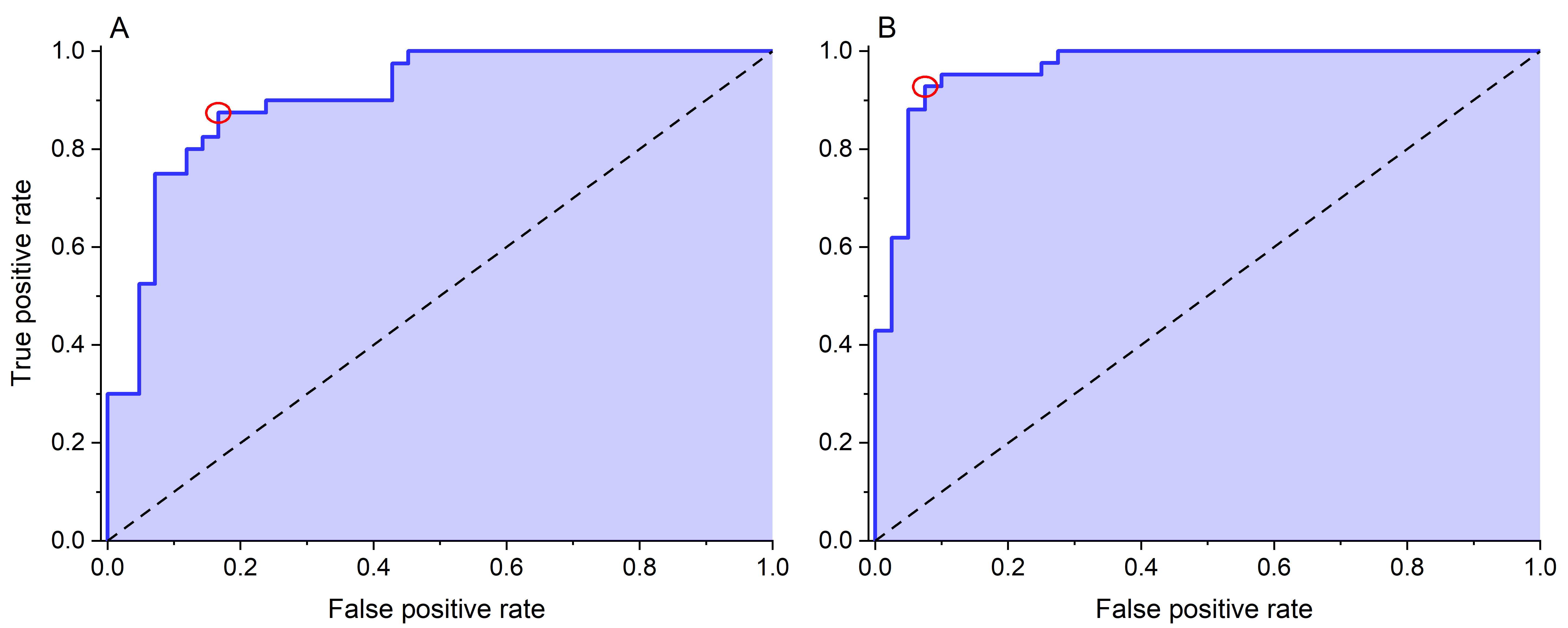

Supplement: Supplementary file 2 — Figure S2. Receiver operating characteristic (ROC) curves to investigate discrimination accuracy between essential tremor (ET) and dystonic tremor (DT) according to peak prominence (A) and peak width (B). Red circles indicate optimal operating points (0.167, 0.875 for peak prominence; 0.075, 0.928 for peak width). [file MDS-40-2654-s004.tif]
