# Supplementary material for: Defining the Rhythm: Developing a New Method to Describe Tremor and Myoclonus
Source: Mov Disord. 2025 Sep 9;40(12):2654–63. doi: 10.1002/mds.70034 (PMC12710117; doi:10.1002/mds.70034)
Supplement: Supplementary file 4 — Data S1. Supporting Information. [file MDS-40-2654-s002.docx]

Supplementary materials and methods

Patients

Patients were retrospectively selected from two sources: the movement disorders analysis clinic and the research lab. In the clinical setting, patients were referred for routine diagnostic purposes, specifically to assess the electrophysiological characteristics of their movement disorder to support diagnosis. From this database, we identified individuals with a final diagnosis of tremor or myoclonus. In parallel, we included patients from the research lab who had previously participated in studies involving myoclonus and various forms of tremor. Only those patients whose diagnoses were both clinically (based on a review of medical records and neurological examinations) and electrophysiologically confirmed according to current diagnostic criteria were included in the present study ^1, 2^. None of the patients with cortical myoclonus exhibited cortical tremor and patients with dystonic tremor all showed dystonic hand tremor, with both tremor and dystonia affecting the same body part. None of the tremors were task specific. Patients were under regular treatment at the time of testing.

EMG recording

EMG were recorded from a single channel by means of 10/20 mm diameter Ag/AgCl cup/pre-gelled adhesive electrodes, sampled at 5 kHz and bandpass filtered (0.5-2 kHz) with a CED 1401 A/D laboratory interface (Cambridge Electronic Design, Cambridge, UK) or Micromed SD Flexi Plus laboratory interface (Micromed S.p.A., Mogliano Veneto, Italy). Electrodes were placed on the muscle most visibly affected by tremor or myoclonus, arranged in a belly-tendon montage. Muscle activity recordings were obtained under conditions that maximally activated it: standing for orthostatic tremor (OT), holding arms outstretched (hands prone) for essential tremor (ET) and dystonic tremor (DT), and during specific activation conditions associated with myoclonus. All patients with myoclonus were evaluated during postural activation, as action-induced myoclonus was their most prominent clinical feature. None of the assessments were conducted at rest or during sensory stimulation. Each recording lasted approximately 90 seconds (between 80 and 100 seconds) of continuous postural holding, without any alternating rest periods. The analysis included the entire recording. The longer duration was chosen to provide more comprehensive information about the tremor or myoclonus pattern, while ensuring that patients did not experience fatigue. Offline processing of signals included band-pass filtering between 1 and 1000 Hz and band-stop filtering between 48 and 52 Hz using a zero-phase, fourth-order Butterworth filter.

PSD peaks and harmonics interpolation

To calculate peak prominence and the PB ratio, the EMG power underlying the tremor-related peak was estimated by interpolating the PSD after removing the peak and its surrounding values. Specifically, 15 data points (each corresponding to 0.1 Hz) on either side of the peak frequency were excluded, and the resulting gap was interpolated using the MATLAB function ‘interp1’ with the ‘pchip’ (piecewise cubic Hermite interpolating polynomial) method. The PSD value at the peak frequency was then extracted from the interpolated spectrum. The same approach was applied to interpolate the main peak and its harmonics. In all cases, visual inspection of the PSD was performed before and after interpolation to confirm the validity of the procedure.

Power spectral density analysis in healthy controls

To investigate the possible contribution of voluntary muscle activity associated with posture to the power spectral density (PSD) observed in myoclonus, we compared these data to PSDs obtained from surface electromyography in 72 healthy subjects. Recordings were taken from the right extensor carpi radialis muscle while subjects maintained an outstretched arm posture (hands prone), for approximately 90 seconds (between 80 and 100 seconds). PSDs were computed using the same procedure described in the main text. Statistical comparisons were performed across the full spectrum (0.1–50 Hz) using a non-parametric, cluster-based permutation test. Group-mean differences were compared against a null distribution generated by randomly shuffling condition labels over 1,000 permutations. For each permutation, point-wise differences were z-scored and thresholded (two-tailed p < 0.05); clusters of contiguous supra-threshold points were then identified using MATLAB’s bwconncomp function. The largest cluster from each permutation defined the null distribution of cluster sizes. Observed group differences were processed in the same way, and clusters smaller than the 95th percentile of the null distribution were excluded. Final p-values were derived from the standard normal cumulative distribution. This approach controls for multiple comparisons without relying on parametric assumptions.

Supplementary results

When peak detection was applied to the entire 1-48 Hz spectrum rather than restricted to phenotype-specific frequency ranges, the largest peak was identified at >12 Hz in 18/40 ET patients and 24/42 DT patients, while no OT patients (0/36) had their largest peak >20 Hz. These peaks likely represent high-amplitude, non–tremor-related EMG activity and illustrate the importance of diagnostic context in spectral analysis.

Supplementary Figure 1 shows the PSDs of healthy subjects and patients with myoclonus, along with their statistical comparison. A significant cluster was identified in the 1.7–39.8 Hz frequency range, in which patients with myoclonus exhibited significantly higher power values (mean p-value: 0.00035).

To further explore the possibility to discriminate ET and DT by focusing on spectral features showing statistically significant differences between these two groups (peak prominence and peak width), we performed a post hoc receiver operating characteristic (ROC) analysis comparing the two groups separately for both variables, using ET as the positive class. This analysis, shown in the Supplementary Figure 2, yielded high classification accuracy for both variables (area under the curve - AUC - 0.905 and 0.963 for peak prominence and peak width, respectively). The optimal cut-off value for peak prominence, identified using Youden’s index, was 6.53. This threshold yielded a sensitivity of 87.5% and a specificity of 83.3%. The optimal cut-off value for peak width, identified using Youden’s index, was 1.76 Hz. This threshold yielded a sensitivity of 92.8% and a specificity of 92.5%.

A similar analysis was run to explore the discriminative value of peak width between DT and myoclonus, as these two groups showed the closest overlap in peak width distribution (Supplementary Figure 3). In this analysis, DT was set as the positive class. The area under the curve was 0.78, indicating moderate/good discriminative ability. The optimal cut-off value for peak width, identified using Youden’s index, was 2.21 Hz. This threshold yielded a sensitivity of 100% and a specificity of 88.57%.

Figure captions

Supplementary figure 1: panels A and B show the power spectral densities (PSDs) of individual subjects in the myoclonus and healthy control groups, respectively. For visual comparison, voltage values were converted to Z-scores and baseline offsets were removed. Each line represents a single subject. Panel C displays the group-averaged PSDs (blue: myoclonus; green: healthy controls), with shaded areas indicating the standard error of the mean. The horizontal black line marks the frequency range in which a statistically significant difference between groups was identified.

Supplementary figure 2. ROC curves to investigate discrimination accuracy between ET and DT according to peak prominence (panel A) and peak width (panel B). Red circles indicate optimal operating points (0.167, 0.875 for peak prominence; 0.075, 0.928 for peak width).

Supplementary figure 3. ROC curves to investigate discrimination accuracy between DT and myoclonus according to peak width. The red circle indicates the optimal operating point (1, 0.875).

References

1. Bhatia KP, Bain P, Bajaj N, et al. Consensus Statement on the classification of tremors. from the task force on tremor of the International Parkinson and Movement Disorder Society. Mov Disord 2018;33(1):75-87.

2. Latorre A, van der Veen S, Pena A, et al. IAPRD new consensus classification of myoclonus. PARKINSONISM & RELATED DISORDERS 2024.
