# Supplementary material for: Defining the Rhythm: Developing a New Method to Describe Tremor and Myoclonus
Source: Mov Disord. 2025 Sep 9;40(12):2654–63. doi: 10.1002/mds.70034 (PMC12710117; doi:10.1002/mds.70034)
Supplement: Supplementary file 5 — Table S1. Supplementary Table. [file MDS-40-2654-s003.docx]

Supplementary Table.

| **Measure** | **OT**  **(mean ± SD)** | **ET**  **(mean ± SD)** | **DT**  **(mean ± SD)** | **Myoclonus**  **(mean ± SD)** |
| --- | --- | --- | --- | --- |
| **Peak Prominence** | 65.17 ± 6.24 | 21.17 ± 4.42 | 3.77 ± 0.53 | 1.62 ± 0.09 |
| **PB Ratio** | 37.34 ± 5.58 | 7.10 ± 2.26 | 4.49 ± 0.70 | 1.69 ± 0.06 |
| **Peak Frequency** | 14.53 ± 0.21 | 5.51 ± 0.20 | 5.56 ± 0.22 | 20.62 ± 1.28 |
| **Peak Width** | 0.99 ± 0.01 | 1.10 ± 0.02 | 3.94 ± 0.26 | 18.45 ± 2.41 |
